# Psychometric properties of the 11-item Trust in the Medical Profession scale (TIMP) Malay version

**DOI:** 10.1186/s41687-026-01139-5

**Published:** 2026-07-08

**Authors:** Asrul Amirullah, Noorazrul Yahya, Hanani Abdul Manan

**Affiliations:** 1 Centre of Diagnostic, Therapeutic and Investigative Sciences (CODTIS), Faculty of Health Sciences, National University of Malaysia, Kuala Lumpur, Malaysia; 2 Department of Radiology, Faculty of Medicine, National University of Malaysia, Kuala Lumpur, Malaysia

**Keywords:** Trust, Physicians, Psychometrics, Reproducibility of results, Factor analysis, Statistical, Malaysia

## Abstract

**Background:**

Trust in the medical profession is important for patient engagement, treatment adherence, and effective healthcare relationships; thus, a culturally appropriate patient-reported instrument for measuring this construct in Malaysia is needed. This study aimed to translate and culturally adapt the 11-item Trust in the Medical Profession scale (TIMP) into Malay and evaluate its psychometric properties in a Malaysian sample.

**Methods:**

A cross-sectional psychometric study was conducted among 376 Malaysian adults with prior COVID-19 care experience. Content validity was evaluated by a six-expert panel. Reliability was assessed using Cronbach’s alpha and McDonald’s omega. Structural validity was examined using exploratory factor analysis (EFA) and confirmatory factor analysis (CFA). Convergent validity was evaluated through correlations with related constructs, namely the *Skala Kepuasan Interaksi Perubatan* (SKIP), the Malay Patient Measure of Safety-10 (M-PMOS-10), and the Malay version of the Multidimensional Scale of Perceived Social Support (MSPSS-M).

**Results:**

The mean TIMP score was 3.02 (SD = 0.94) on a 5-point scale. Content validity was strong (I-CVI = 0.83–1.00; S-CVI/Ave = 0.97; mean κ = 0.97). Reliability was excellent (α = 0.94, 95% CI [0.93, 0.95]; ω = 0.94, 95% CI [0.93, 0.95]). EFA supported one factor (KMO = 0.963; Bartlett’s χ²(55) = 2611.91, *p* < .001; first eigenvalue = 6.84, 62.1% variance) with loadings from 0.75 to 0.80. The one-factor CFA fit the data well (χ²(44) = 73.52, *p* = .003; CFI = 0.989; TLI = 0.986; RMSEA = 0.042 [90% CI 0.024–0.059]; SRMR = 0.023), with CR = 0.944 and AVE = 0.585. Convergent validity was supported by positive correlations with SKIP (*r* = .62, *p* < .001), M-PMOS-10 (*r* = .68, *p* < .001), and MSPSS-M (*r* = .16, *p* = .002).

**Conclusions:**

The Malay TIMP demonstrated strong psychometric properties in this sample and provides initial evidence for assessing trust in the medical profession among Malay-speaking Malaysian adults. Further validation in broader community and clinical samples is warranted.

## Highlights

The Malay TIMP provides initial psychometric evidence for a Malay-language measure of general trust in the medical profession.

Content validity indices and modified kappa values indicated strong expert agreement across all 11 items.

EFA and CFA confirmed a robust unidimensional structure with excellent internal consistency reliability.

Positive correlations with SKIP, M-PMOS-10, and MSPSS-M supported the convergent validity of the Malay TIMP.

## Introduction

Trust in healthcare is a central component of effective professional relationships and has important implications for care-seeking, communication, and adherence to medical advice [13]. Trust is also relevant to patient-centred outcomes because it reflects how patients evaluate healthcare relationships, professional credibility, and the willingness to depend on medical expertise in situations of uncertainty.

Trust is recognized as a foundational element of healthcare quality and ethics. Modern reviews note that trust is a core professional norm in nursing and medicine, serving to overcome the inherent power asymmetry between providers and patients [13]. It has a normative value and a moral dimension, characterized by confident reliance on providers’ competence and goodwill [13]. Because patients often must submit to care they do not fully control, trust is essential. When trust is present, it is associated with better engagement and adherence; when trust is lacking, patients may delay care or disregard medical advice. Therefore, validly measuring trust can inform interventions to improve healthcare relationships and outcomes. Yet, despite growing recognition of trust in the medical profession as a meaningful patient-centred construct, its measurement in Malaysia remains insufficiently established. The absence of a culturally adapted and psychometrically evaluated Malay-language instrument limits the ability to assess this construct rigorously in local research and practice. As interest in relational aspects of care and patient-centred outcomes continues to expand, the validation of a suitable Malay-language measure is therefore needed.

Many studies have emphasized that trust is a multifaceted construct that may vary across cultural and healthcare contexts. Instruments that assess trust should therefore distinguish between trust in medicine generally and trust in one’s own doctor. The 11-item Trust in the Medical Profession scale (TIMP) was originally developed to measure general trust in physicians [10]. Before it can be used in a new sociocultural setting such as Malaysia, its relevance, reliability, and internal structure must be empirically established. We follow the example of other Malay-language instrument adaptations (e.g., for patient safety culture) by conducting a full evaluation of translation adequacy, content validity, factor structure, reliability, and convergent validity. Valid measurement of this construct requires careful instrument development and evaluation. Best practices in scale development demand thorough content validation by experts and transparent reporting of psychometric testing [9, 13]. In particular, guidelines note that with six experts, an item-level content validity index (I-CVI) of ≥ 0.78 is considered acceptable, and a scale-average CVI (S-CVI) of ≥ 0.90 indicates excellent content validity [9]. A strong validity argument for a scale also includes demonstrating that the items form a coherent factor (using exploratory factor analysis [EFA] and confirmatory factor analysis [CFA]) and yield high internal consistency [8, 9]. The original TIMP [10] contains 11 statements about physicians and medicine, rated on a 5-point Likert scale (1 = strongly disagree to 5 = strongly agree). Example items include statements such as “Medical doctors’ decisions are based on sound reasoning” (adapted from patient trust literature) and “Overall, doctors can be trusted.” Higher mean scores indicate greater trust. In Hall et al.,’s work, the TIMP formed a single-factor scale with good internal consistency (α = 0.89) [10].

Despite the importance of trust in healthcare systems, there is currently limited validated instrumentation for measuring general trust in the medical profession in the Malaysian context. Cultural norms, healthcare structures, and communication patterns may influence how trust in physicians is conceptualized and expressed. Instruments developed in Western settings cannot be assumed to retain their validity when applied in different sociocultural environments without empirical testing. Therefore, rigorous translation and psychometric evaluation are necessary before such tools can be used confidently in Malaysian research and practice. This is particularly relevant in populations with recent complex healthcare experiences, such as individuals recovering from COVID-19, among whom persistent neurological and cognitive sequelae may influence continuing engagement with healthcare systems and professionals [16, 17]. Establishing the validity and reliability of a Malay-language version of the TIMP would enable systematic assessment of trust in the medical profession and support research aimed at improving patient–provider relationships within Malaysia’s healthcare system. In the Malaysian context, such patient-centred assessment is especially important because healthcare experiences are embedded within broader family, social, and caregiving responsibilities that may shape how people engage with health services [4]. This study aimed to translate and culturally adapt the 11-item TIMP into the Malay language and evaluate its content validity, factor structure, reliability, and convergent validity among Malaysian adults.

## Methods

### Study procedure

The study was conducted in several phases. It began with the translation and cultural adaptation of the TIMP [10], followed by content validation by an expert panel. Subsequently, psychometric evaluation was performed using survey data to examine the internal structure, reliability, and convergent validity of the Malay version of the Trust in the Medical Profession scale (TIMP).

### Phase 1: Translation of the TIMP

The translation process followed a structured cross-cultural adaptation approach to ensure that the Malay version retained conceptual, semantic, and contextual equivalence with the original English instrument [22]. Permission to use and translate the instrument was obtained from the original source before the translation process began.

The forward translation involved translating the original English TIMP into Malay by two independent bilingual translators: one with health or medical research expertise and one with linguistic expertise. This step was intended to identify differences in wording, detect ambiguities, and generate alternative phrasings where necessary. The two forward-translated versions were then compared and reconciled by the research team to produce a single preliminary Malay version. Reconciliation focused on ensuring that the selected wording was conceptually accurate, culturally appropriate, and easily understood by Malay-speaking respondents.

The reconciled Malay version was subsequently back-translated into English by two independent bilingual translators who were not involved in the forward translation process and were blinded to the original English instrument. The purpose of back translation was to evaluate whether the translated Malay items preserved the original meaning of the TIMP items. A review process was then conducted in which the back-translated versions were compared with the original English scale to identify discrepancies in wording, meaning, and conceptual interpretation. Any inconsistencies were discussed and resolved by the research team to improve equivalence between the source and target versions.

Following this process, the preliminary Malay TIMP was submitted for expert review to assess the relevance and appropriateness of each translated item. This step formed the basis of the content validation phase and ensured that the final Malay version was suitable for psychometric evaluation in the target population.

### Phase 2: Content validation

For content validation, six experts evaluated the Malay version of the TIMP. The expert panel consisted of members with backgrounds in medicine, psychology, linguistics, and translation. This multidisciplinary composition was considered appropriate because the TIMP requires evaluation of healthcare relevance, psychological interpretation of trust, Malay-language clarity, and cross-cultural translation equivalence. Each expert reviewed the relevance of every item to the construct being measured using a 4-point ordinal rating scale. The content validity index (CVI) was then computed. Prior to the calculation of CVI, item ratings were recoded into dichotomous values, whereby ratings of 3 or 4 were coded as 1, indicating that the item was quite relevant or highly relevant, whereas ratings of 1 or 2 were coded as 0, indicating that the item was not relevant [24]. The number of experts in agreement for each item was determined by summing the relevant ratings across the six experts.

The Item-Level Content Validity Index (I-CVI) was calculated by dividing the number of experts in agreement by the total number of experts. For a six-member expert panel, an I-CVI value of at least 0.78 is generally considered acceptable [9, 14]. In addition, the Scale-Level Content Validity Index using the averaging method (S-CVI/Ave) was calculated as the mean of all I-CVI values across the items. An S-CVI/Ave value of 0.90 or higher indicates excellent content validity [9]. To further account for chance agreement, the modified kappa coefficient was also computed for each item. Following the expert review, the panel discussed items with borderline ratings and suggested wording refinements where necessary; however, no items were deleted.

### Phase 3: Pilot testing, construct validation, and convergent validity

A total of 15 respondents from the target population who fulfilled the study criteria were recruited for pilot testing. The pilot study was conducted to evaluate the comprehensibility of the translated items, the suitability of the questionnaire format, and the feasibility of questionnaire administration and data handling procedures. This phase also provided preliminary evidence regarding item clarity and respondent acceptability. Based on the pilot findings, minor refinements were made to improve the presentation and wording of the Malay TIMP prior to the main study. Descriptive statistics were used in the pilot phase, with categorical variables presented as frequency and percentage, and continuous variables presented as mean with standard deviation (SD) or median with interquartile range (IQR), depending on data distribution.

Construct validation was subsequently conducted using data from 376 respondents. The analysis included both exploratory factor analysis (EFA) and confirmatory factor analysis (CFA). EFA was first performed to identify the latent structure of the Malay TIMP and to determine whether the translated version retained the expected dimensionality in the Malaysian context. The suitability of the data for factor analysis was evaluated using the Kaiser–Meyer–Olkin (KMO) measure of sampling adequacy and Bartlett’s test of sphericity. Factor retention was informed by parallel analysis, eigenvalues, and factor loading patterns. A factor loading of greater than 0.40 was considered acceptable.

CFA was then performed to test the adequacy of the hypothesized one-factor model. Model fit was evaluated using the chi-square statistic (χ²), degrees of freedom (df), Comparative Fit Index (CFI), Tucker–Lewis Index (TLI), Root Mean Square Error of Approximation (RMSEA) with 90% confidence interval (CI), and Standardized Root Mean Square Residual (SRMR). Model fit was considered acceptable when SRMR was ≤ 0.08, RMSEA was ≤ 0.08, CFI was > 0.90, and TLI was > 0.95 [12]. Standardized factor loadings, residuals, and modification indices were also examined.

Internal consistency reliability was assessed using Cronbach’s alpha (α) and McDonald’s omega (ω), with 95% bootstrap confidence intervals reported for both indices. Corrected item-total correlations were further examined to evaluate item homogeneity.

Convergent validity was assessed using Pearson correlation coefficients between TIMP and three related measures: the *Skala Kepuasan Interaksi Perubatan* (SKIP), the Malay Patient Measure of Safety-10 (M-PMOS-10), and the Malay version of the Multidimensional Scale of Perceived Social Support (MSPSS-M). Positive associations were expected, with stronger correlations anticipated between TIMP and healthcare-related constructs, particularly SKIP [1]d PMOS-10 [18], and comparatively weaker correlations expected with MSPSS-M [19].

### Study design, population, and eligibility criteria

#### Study site, duration, and sampling method

This study was conducted in hospitals and clinics in Selangor and Kuala Lumpur between November 2025 and March 2026. Participants were recruited using a non-probability sampling approach through in-person contact. Eligible respondents were approached until the required sample for psychometric validation was achieved.

#### Sample size calculation

The sample size was determined based on recommendations for factor analysis and psychometric validation, which commonly suggest a minimum of 10 participants per item for factor analytic procedures [3]. For an 11-item instrument, this subject-to-item ratio would require at least 110 respondents. The final sample of 376 exceeded this minimum and was considered adequate for exploratory factor analysis, confirmatory factor analysis, and reliability estimation.

This study was a cross-sectional psychometric validation study conducted among adults with prior COVID-19 care experience. The reference population comprised Malaysian adults with prior COVID-19 care experience, whereas the target population consisted of Malay-speaking adults who were eligible to complete the Malay version of the TIMP. Eligibility was established through screening items confirming adulthood, residence in Malaysia, and prior COVID-19 care experience. No specific time limit was imposed regarding the date of COVID-19 infection or the recency of last healthcare contact because these data were not collected. Therefore, prior COVID-19 care experience should be interpreted as a sampling characteristic rather than evidence of recent healthcare contact for all participants. Follow-up care status was described separately in the Results section. The study was intended as an initial psychometric validation rather than a nationally representative survey of all Malaysian adults.

The inclusion criteria were adults aged 18 years and above, Malaysian or residing in Malaysia, able to read and understand the Malay language, reporting prior COVID-19 care experience, and willing to participate in the study. Individuals who refused participation, were unable to understand Malay, had significant cognitive impairment based on researcher judgement, or were otherwise unable to complete the questionnaire independently were excluded. Data were collected through a self-administered questionnaire that included demographic items, COVID-19 care pathway questions, and multi-item psychometric measures. Respondents provided informed consent before completing the questionnaire.

### Research tools

Apart from the Malay version of the Trust in the Medical Profession scale (TIMP), this study also used a sociodemographic proforma and three additional instruments for convergent validity assessment.

A study proforma was used to obtain respondents’ sociodemographic information, including age, gender, ethnicity, education level, marital status, employment status, and household income. The TIMP was the main instrument evaluated in this study. The original scale was developed to assess general trust in physicians and consists of 11 items reflecting domains such as competence, honesty, fidelity, and global trust [10]. Items are rated on a 5-point Likert scale, with higher scores indicating greater trust. The original version demonstrated a single-factor structure and good internal consistency, with a Cronbach’s alpha of 0.89 [10].

The *Skala Kepuasan Interaksi Perubatan*-11 (SKIP) [1] was used to assess patient satisfaction with interpersonal aspects of doctor-patient communication. The validated Malay version consists of 11 items and demonstrated a three-factor model with acceptable model fit indices (χ² = 65.805, df = 32, TLI = 0.902, CFI = 0.927, RMSEA = 0.061, SRMR = 0.058) [1]. Composite reliability values ranged from 0.541 to 0.760 [1].

The Malay Patient Measure of Safety-10 (M-PMOS-10) [18] was used to assess patients’ perceptions of safety-related factors in healthcare interactions. The instrument contains 10 items rated on a 5-point Likert scale. The validated Malay version demonstrated excellent internal consistency, with Raykov’s rho = 0.929, and confirmatory factor analysis supported a revised single-factor structure with acceptable fit [18].

The Malay version of the Multidimensional Scale of Perceived Social Support (MSPSS-M) [19] was used to assess perceived support from family, friends, and significant others. This instrument contains 12 items and demonstrated good psychometric properties, including Cronbach’s alpha of 0.89, parallel-form reliability of 0.94, and test-retest reliability of 0.77 [19]. Factor analysis supported a three-factor structure, with the factors accounting for 75.9% of the variance [19].

## Results

### Participant characteristics

**Fig. 1:**
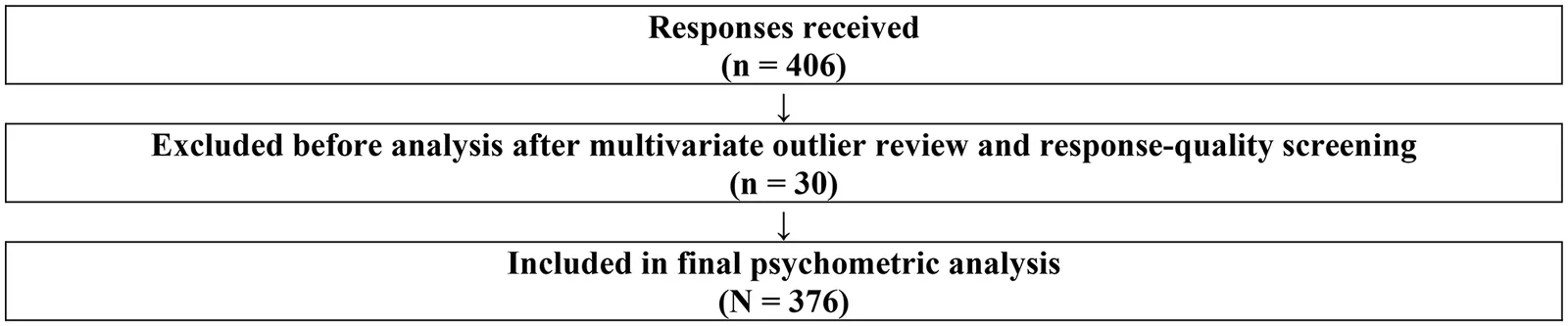
Participant recruitment flow diagram

As shown in Fig. 1, a total of 406 responses were received. Thirty responses were excluded before analysis based on multivariate outlier review and response-quality screening, resulting in a final analytic sample of 376 respondents. There were no missing data for the demographic variables. Participants ranged in age from 19 to 74 years (M = 41.51, SD = 10.84). The sample comprised 196 men (52.1%) and 180 women (47.9%).

The sample was diverse in terms of marital status, education, employment, income, and COVID-19 care pathways. Most respondents were married (62.0%), followed by single (30.1%), divorced or separated (4.5%), and widowed (3.5%). In terms of educational attainment, the largest proportion had completed upper secondary education (28.2%), followed by a bachelor’s degree (24.2%) and diploma-level education (18.4%).

Regarding employment status, more than half of the respondents were in full-time employment (54.5%), followed by self-employment (12.2%), retirement (7.7%), unemployment (7.4%), and homemaker status (7.2%). In terms of monthly household income, the largest group fell within the RM2,500 to RM4,849 category (31.6%), followed by RM4,850 to RM10,959 (29.0%) and below RM2,500 (21.0%).

With respect to initial COVID-19 care, the largest proportion reported home quarantine or home monitoring (42.8%), followed by general hospital ward care (28.7%), quarantine centre or PKRC care (23.1%), and intensive care unit care (5.3%). For follow-up care, 43.1% received care exclusively from public health facilities, 27.9% attended both public and private facilities, 16.2% used private facilities only, and 12.8% reported no regular follow-up care (see Table 1).

**Table 1:** Respondent demographics

Variable	Category	*n*	%	Mean (SD) / Range
Age (years)				41.51 (10.84); 19–74
Gender	Male	196	52.1	
	Female	180	47.9	
Marital status	Married	233	62.0	
	Single	113	30.1	
	Divorced/Separated	17	4.5	
	Widowed	13	3.5	
Highest education	Doctorate	2	0.5	
	Master’s degree	31	8.2	
	Bachelor’s degree	91	24.2	
	Diploma	69	18.4	
	Upper secondary	106	28.2	
	Lower secondary	49	13.0	
	Primary school	24	6.4	
	No formal education	4	1.1	
Employment status	Full-time employment	205	54.5	
	Self-employed	46	12.2	
	Retired	29	7.7	
	Unemployed	28	7.4	
	Homemaker	27	7.2	
	Part-time employment	22	5.9	
	Student	19	5.1	
Monthly household income	< RM2,500	79	21.0	
	RM2,500-RM4,849	119	31.6	
	RM4,850-RM10,959	109	29.0	
	RM10,960-RM15,039	27	7.2	
	≥ RM15,040	16	4.3	
	Prefer not to disclose	26	6.9	
Initial COVID-19 care	Home quarantine/home monitoring	161	42.8	
	General hospital ward	108	28.7	
	Quarantine centre/PKRC	87	23.1	
	Intensive care unit	20	5.3	
Follow-up care facility	Public facility only	162	43.1	
	Combination of public and private facilities	105	27.9	
	Private facility only	61	16.2	
	No regular follow-up care	48	12.8	

### Content validity

All 11 Malay items demonstrated acceptable-to-excellent relevance ratings by the six experts. The item-level content validity indices (I-CVI) ranged from 0.83 to 1.00 (Table 2), all exceeding the minimum recommended value of 0.78 for panels of this size [20, 25]. Likewise, the scale-level content validity index (S-CVI/Ave) was 0.97, well above the conventional 0.90 threshold for excellent content validity [20]. The modified Cohen’s kappa coefficients (κ*), which adjust for chance agreement, were also very high (range 0.81–1.00, mean 0.97), indicating almost perfect inter-expert agreement. Values greater than 0.74 are generally considered excellent [25]. In summary, these content-validity results indicate that the experts generally found the Malay trust items to be clear, relevant, and representative of the doctor-patient trust construct.

**Table 2:** TIMP content validity

Item	Item Text	I-CVI	κ* (modified kappa)
1	Doctors in [general] care about their patients’ health just as much or more as their patients do. *Secara umumnya*,* doktor mengambil berat tentang kesihatan pesakit sama seperti pesakit itu sendiri.*	1.00	1.00
2	Sometimes doctors care more about what is convenient for them than about their patients’ medical needs. *Ada kalanya doktor lebih mengutamakan apa yang mudah bagi mereka berbanding keperluan perubatan pesakit.*	0.83	0.81
3	Doctors are extremely thorough and careful *Doktor sangat teliti dan berhati-hati.*	1.00	1.00
4	You completely trust doctors’ decisions about which medical treatments are best *Anda percaya sepenuhnya kepada doktor untuk mendapatkan rawatan yang terbaik.*	1.00	1.00
5	Doctors are totally honest in telling their patients about all of the different treatment options available for their conditions *Doktor jujur dalam memberitahu pesakit tentang pilihan rawatan yang berbeza-beza untuk keadaan pesakit.*	1.00	1.00
6	Doctors think only about what is best for their patients. *Doktor hanya mempertimbangkan kaedah rawatan yang terbaik untuk pesakit mereka.*	1.00	1.00
7	Sometimes doctors do not pay full attention to what patients are trying to tell them. *Ada kalanya doktor kurang memberi perhatian terhadap apa yang ingin disampaikan oleh pesakit.*	0.83	0.81
8	Doctors always use their very best skill and effort on behalf of their patients. *Doktor sentiasa menggunakan kemahiran dan usaha yang terbaik untuk pesakit.*	1.00	1.00
9	You have no worries about putting your life in the hands of doctors. *Anda berasa yakin menerima rawatan di bawah jagaan doktor.*	1.00	1.00
10	A doctor would never mislead you about anything. *Seorang doktor tidak akan sesekali memberikan maklumat yang salah kepada anda dalam setiap perkara.*	1.00	1.00
11	All in all, you trust doctors completely. *Secara keseluruhan*,* anda mempunyai tahap kepercayaan yang tinggi terhadap doktor.*	1.00	1.00
**Scale-level indices**		**S-CVI/Ave = 0.97**	Mean κ* = 0.97

Note. I-CVI = item-level content validity index; κ* = modified chance-adjusted kappa. All I-CVI values exceed the 0.78 guideline for six experts [20, 25]. The S-CVI/Ave (average of I-CVIs) of 0.97 exceeds the conventional 0.90 criterion [20], indicating excellent overall content validity

### Descriptive statistics of items

At the item level, mean scores for the Malay TIMP ranged from 2.97 to 3.13, with standard deviations ranging from 1.12 to 1.24. The highest mean was observed for TIMP_09 (*M* = 3.13, *SD* = 1.12), whereas the lowest means were observed for TIMP_06 and TIMP_08 (both *M* = 2.97). Median scores were 3.00 for all items, and responses spanned the full range of the 5-point scale (see Table 3).

**Table 3:** TIMP item descriptive statistics

Item	M	SD	Median	Min	Max
TIMP_01	3.02	1.21	3.00	1	5
TIMP_02 (rev)	3.01	1.23	3.00	1	5
TIMP_03	3.05	1.22	3.00	1	5
TIMP_04	3.01	1.15	3.00	1	5
TIMP_05	3.05	1.24	3.00	1	5
TIMP_06	2.97	1.14	3.00	1	5
TIMP_07 (rev)	2.99	1.21	3.00	1	5
TIMP_08	2.97	1.21	3.00	1	5
TIMP_09	3.13	1.12	3.00	1	5
TIMP_10	3.06	1.18	3.00	1	5
TIMP_11	3.01	1.16	3.00	1	5

Note. TIMP_02 and TIMP_07 are negatively worded items and were reverse scored before computing item descriptives and total TIMP scores; higher values therefore consistently indicate higher trust

### Construct validity and reliability

Construct validity. The suitability of the data for factor analysis was supported by a Kaiser-Meyer-Olkin value of 0.963 and a significant Bartlett’s test of sphericity, χ²(55) = 2611.91, *p* < .001. Exploratory factor analysis supported a one-factor solution, with the first factor yielding an eigenvalue of 6.84 and accounting for 62.15% of the total variance. As shown in Table 4, all items loaded strongly on the single factor, with factor loadings ranging from 0.75 to 0.80, communalities ranging from 0.56 to 0.64, and uniqueness values ranging from 0.36 to 0.44.

Confirmatory factor analysis further supported the one-factor structure of the Malay TIMP. The model demonstrated good fit to the data, χ²(44) = 73.52, *p* = .003, CFI = 0.989, TLI = 0.986, RMSEA = 0.042 (90% CI [0.024, 0.059]), and SRMR = 0.023. Standardized factor loadings ranged from 0.75 to 0.80, with standardized error variances ranging from 0.36 to 0.44 (Table 4). In addition, the model showed strong composite reliability (CR = 0.944) and acceptable average variance extracted (AVE = 0.585), supporting the convergent representation of the latent construct (Table 5).

**Table 4:** TIMP EFA and CFA item results

Item	EFA loading	h²	u²	CFA loading	CFA error
TIMP_01	0.75	0.56	0.44	0.75	0.44
TIMP_02 (rev)	0.78	0.61	0.38	0.78	0.38
TIMP_03	0.76	0.57	0.43	0.76	0.43
TIMP_04	0.75	0.56	0.43	0.75	0.43
TIMP_05	0.80	0.64	0.36	0.80	0.36
TIMP_06	0.75	0.57	0.43	0.75	0.43
TIMP_07 (rev)	0.76	0.57	0.43	0.76	0.43
TIMP_08	0.76	0.58	0.42	0.76	0.42
TIMP_09	0.76	0.57	0.42	0.76	0.42
TIMP_10	0.76	0.57	0.43	0.76	0.43
TIMP_11	0.78	0.60	0.40	0.78	0.40

Reliability. The Malay TIMP demonstrated excellent internal consistency, with Cronbach’s alpha of 0.939 and McDonald’s omega of 0.939 (Table 5).

**Table 5:** TIMP model-level psychometric indices

Index	Value
KMO	0.963
Bartlett’s test of sphericity, χ²(55)	2611.91*
First eigenvalue	6.84
Variance explained (%)	62.15
χ²(44)	73.52
*p* value for χ²	0.003
CFI	0.989
TLI	0.986
RMSEA	0.042
90% CI for RMSEA	0.024–0.059
SRMR	0.023
Composite reliability (CR)	0.944
Average variance extracted (AVE)	0.585
Cronbach’s alpha (α)	0.939
McDonald’s omega (ω)	0.939

Note. KMO = Kaiser-Meyer-Olkin measure of sampling adequacy; CFI = Comparative Fit Index; TLI = Tucker-Lewis Index; RMSEA = Root Mean Square Error of Approximation; CI = confidence interval; SRMR = Standardized Root Mean Square Residual; CR = composite reliability; AVE = average variance extracted

*p* < .001 for Bartlett’s test of sphericity

### Convergent validity

Correlation analyses supported theoretical predictions (Table 6). The TIMP mean score correlated positively with SKIP, which measures satisfaction with doctor-patient interaction (*r* = .62, *p* < .001), and with M-PMOS-10, which measures patients’ perceptions of safety-related factors in healthcare interactions (*r* = .68, *p* < .001). By contrast, the correlation between TIMP and MSPSS-M was small (*r* = .16, *p* = .002). This pattern suggests that general trust in the medical profession was more strongly related to healthcare-proximal constructs than to broader perceived social support, supporting convergent validity (see Table 6).

**Table 6:** Correlations among TIMP and related constructs

Variable	M	SD	TIMP	SKIP	M-PMOS-10	MSPSS
TIMP	3.02	0.94	—			
SKIP	3.00	0.96	0.62 (< 0.001)	—		
M-PMOS-10	3.00	0.93	0.68 (< 0.001)	0.55 (< 0.001)	—	
MSPSS	4.58	1.33	0.16 (0.002)	0.24 (< 0.001)	0.23 (< 0.001)	—

Note. Values are Pearson correlation coefficients with p-values in parentheses. TIMP = Trust in the Medical Profession; SKIP = *Skala Kepuasan Interaksi Perubatan*; M-PMOS-10 = Malay Patient Measure of Safety-10; MSPSS-M = Malay version of the Multidimensional Scale of Perceived Social Support

## Discussion

This study translated, culturally adapted, and evaluated the Malay-language Trust in the Medical Profession scale (TIMP) following rigorous psychometric procedures. The Malay TIMP demonstrated excellent reliability and a clear one-factor structure. Cronbach’s alpha (α = 0.939) and McDonald’s omega (ω = 0.939) indicated highly consistent item responses. These reliability estimates are comparable to those reported in other trust validation studies, including an Indian trust in physician scale [7]. Exploratory and confirmatory factor analyses both supported unidimensionality: a single factor accounted for approximately 62% of the variance, all items loaded strongly on that factor, and CFA fit indices were in the excellent range (CFI ≈ 0.99, RMSEA ≈ 0.04). These findings align with prior cross-cultural validations showing that trust in physicians often behaves as a unitary construct [7, 23]. By contrast, multidimensional solutions have been reported in other settings. For example, Aloba et al. [2] identified a two-factor structure in a Nigerian psychiatric sample, suggesting that cultural context, clinical population, or instrument framing may influence the dimensionality of trust.

The construct of trust is central to sociomedical theory. Our results fit the view of trust as a willingness to be vulnerable in a relationship [15, 23]. In healthcare, patients are inherently vulnerable due to illness, and trust in doctors represents the “optimistic acceptance of [that] vulnerable situation” with confidence that the physician will act in the patient’s best interest [15, 23]. This theoretical framing is supported here: all TIMP items reflect facets of physician competence and caring (e.g. thorough examination, clear communication, honesty), and they coalesce into one latent factor of general trust. Moreover, trust in this context influences patient behaviors: past research shows high trust predicts greater adherence, open disclosure, and satisfaction [7, 23]. The observed positive correlations between TIMP and the healthcare-related measures support this interpretation. This is consistent with the broader patient-centred literature, which increasingly recognizes that self-reported perceptions and experiences provide meaningful information about healthcare quality and outcomes [5, 6]. Higher trust was associated with more favourable evaluations of doctor-patient interaction and patient safety. By contrast, the weaker association with perceived social support suggests that trust in the medical profession is a more domain-specific construct rather than a reflection of general psychosocial support. This distinction is also relevant in the Malaysian setting, where social roles and informal support structures may shape health-related experiences without necessarily translating directly into trust in professional care [4].

These psychometric findings also satisfy key aspects of contemporary validation frameworks. According to COSMIN, content validity, structural validity, and internal consistency are central measurement properties in evaluating patient-reported outcome measures [21]. The present study addressed these properties through expert content validation, EFA and CFA, and reliability estimation using both alpha and omega. The translation methodology also supported content validity by ensuring that the Malay TIMP items retained their intended meaning for local respondents. Future work could extend this validation by assessing test-retest reliability and sensitivity to change, thereby further strengthening the measurement evidence for the Malay TIMP.

Comparing with international studies underscores both consistencies and unique contextual factors. The high reliability and single-factor structure resemble the pattern seen in many other countries’ adaptations of trust scales [7]. Qualitative research suggests some trust dimensions (e.g. technical competence, honesty) are universal, while others (e.g. assurance of treatment, respect) may be particularly salient in resource-constrained settings [7]. In this Malaysian sample, all established TIMP items performed well, implying that the core dimensions of physician trust apply here as well. Notably, cultural norms of deference toward authority might encourage patients to trust doctors by default, but only if that trust is reciprocated through transparency and respect [11, 23]. Indeed, recent Malaysian bioethical analyses argue that truthfulness and patient involvement (e.g. open medical records) are crucial for genuine trust [11, 23]. Our results thus fit a broader narrative: trust cannot be assumed, and instruments like the TIMP are needed to measure and monitor it.

Collectively, these findings provide initial psychometric support for the Malay TIMP as a patient-reported instrument for assessing general trust in the medical profession. The evidence from content validation, factor analysis, reliability testing, and convergent validity suggests that the translated instrument captures the intended construct coherently in this sample. The instrument may therefore support future Malaysian research on patient experience, relational quality of care, and trust in medical professionalism, although additional validation in independent and more diverse samples remains necessary.

### Limitations

Several limitations should be acknowledged. First, test-retest reliability was not assessed; therefore, the temporal stability of the Malay TIMP remains to be examined. Second, the use of self-reported questionnaire data may introduce response bias, including social desirability bias. Third, although convergent validity was examined using theoretically related constructs, discriminant validity with clearly unrelated constructs was not assessed. Fourth, exploratory and confirmatory factor analyses were conducted using the same analytic sample; future studies should examine the factor structure in an independent validation sample. Fifth, because respondents were recruited using a non-probability sampling approach from hospitals and clinics in Selangor and Kuala Lumpur, the findings may not fully generalize to all Malay-speaking Malaysian adults or to other regional healthcare settings. Sixth, the sample consisted of adults with prior COVID-19 care experience, and the study did not collect detailed data on the timing of infection or the recency of last healthcare contact. Previous illness experience and healthcare contact may influence trust in the medical profession. Future studies should therefore evaluate the Malay TIMP in broader community and clinical samples, assess temporal stability, and examine discriminant validity and measurement performance across relevant subgroups.

## Conclusion

In summary, the Malay adaptation of the TIMP demonstrated strong content validity, a clear unidimensional factor structure, high internal consistency, and theoretically coherent convergent validity in this sample. These findings provide initial psychometric evidence supporting the Malay TIMP as a patient-reported instrument for assessing general trust in the medical profession among Malay-speaking adults in Malaysia. Further studies are needed to confirm its temporal stability, discriminant validity, and performance in broader community and clinical samples.

## Data Availability

The datasets used and/or analysed during the current study are available from the corresponding author on reasonable request.
